# Nimesulide-induced fatal acute liver failure in an elderly woman with metastatic biliary adenocarcinoma. A case report: Insuficiência hepática aguda fatal induzida por nimesulida em uma mulher idosa com adenocarcinoma biliar metastático. Um relato de caso

**DOI:** 10.1590/1516-3180.2013.7550003

**Published:** 2014-09-19

**Authors:** Sara Santos Bernardes, André Souza-Nogueira, Estefânia Gastaldello Moreira, Marina Okuyama Kishima, Alda Fiorina Maria Losi Guembarovski, Tercilio Luiz Turini, Conceição Aparecida Turini

**Affiliations:** I MSc. Assistant Professor, Department of General Pathology, Universidade Estadual de Londrina (UEL), Londrina, Paraná, Brazil.; II MSc. Universidade Estadual de Londrina (UEL), and Poison Information Center, University Hospital, Universidade Estadual de Londrina, Londrina, Paraná, Brazil.; III PhD. Adjunct Professor, Department of Physiological Sciences, Universidade Estadual de Londrina (UEL), Londrina, Paraná, Brazil.; IV MD. Adjunct Professor, Department of Clinical Medicine, University Hospital, Universidade Estadual de Londrina (UEL), Londrina, Paraná, Brazil.; V MD. Adjunct Professor, Department of Pathology, Clinical and Toxicology Analysis, Universidade Estadual de Londrina (UEL), and Poison Information Center, University Hospital, Universidade Estadual de Londrina (UEL), Londrina, Paraná, Brazil.

**Keywords:** Anti-inflammatory agents, non-steroidal, Liver failure, acute, Drug-induced liver injury, Cholestasis, Drug toxicity, Anti-inflamatórios não esteroides, Falência hepática aguda, Doença hepática induzida por drogas, Colestase, Toxicidade de drogas

## Abstract

**CONTEXT:**

Nimesulide is a selective inhibitor of the enzyme cyclooxygenase 2. Although considered to be a safe drug, cases of acute hepatitis and fulminant liver failure have been reported in Europe, the United States and South America, especially among elderly female patients. Until now, there had not been any reports in the literature relating to Brazilian subjects.

**CASE REPORT:**

An 81-year old female who had been using nimesulide therapy for six days presented hematemesis and epistaxis two days before hospitalization. Clinical examination showed an extensive coagulation disorder, diffuse hematomas, hypotension and tachypnea. Laboratory tests revealed abnormalities in coagulation tests; leukocytosis; reduced platelet, hemoglobin and red blood cell counts; and elevated direct bilirubin, serum aspartate transaminase (AST), gamma-glutamyl transpeptidase (GGT), alkaline phosphatase and renal function biomarkers. Hepatitis B and C tests were not reactive. Carcinoembryonic antigen (CEA), CA-19-9 and CA-125 levels were increased by, respectively, 1,000, 10,000 and 13 fold, whereas the alpha-fetoprotein level was normal, thus indicating a malignant tumor in the bile duct that did not originate from the liver. Thirty-six hours after hospitalization, the patient’s condition worsened, leading to death. The necropsy findings included acute hepatitis with hepatocellular collapse, as well as metastasis of a carcinoma, probably from the bile duct.

**CONCLUSION:**

Despite the carcinoma presented by the patient, nimesulide use may have contributed towards the fatal acute liver failure. Until this issue has been clarified, caution is required in prescribing nimesulide for liver disease patients.

## RESUMO

**CONTEXTO:**

A nimesulida é um inibidor seletivo da enzima ciclo-oxigenase 2. Apesar de ela ser considerada fármaco seguro, casos de hepatite aguda e falência hepática fulminante foram descritos na Europa, Estados Unidos e América do Sul, principalmente em idosos do sexo feminino. Até o momento não há relatos na literatura em indivíduos brasileiros.

**RELATO DE CASO:**

Mulher de 81 anos, em uso terapêutico de nimesulida por seis dias, apresentou hematêmese e epistaxe dois dias antes da hospitalização. O exame clínico mostrou importante distúrbio de coagulação, hematomas difusos, hipotensão e taquipneia. Os exames laboratoriais mostravam alteração das provas de coagulação, leucocitose, redução do número de plaquetas, hemoglobina e hemácias, aumento de bilirrubina direta, elevação dos valores de aspartato aminotransferase (AST), gama glutamil transpeptidase (GGT), fosfatase alcalina e marcadores de função renal. Exames para hepatite B e C apresentaram-se não reagentes. Elevados níveis dos marcadores antígeno carcinoembriônico (CEA), CA-19-9 e CA-125 foram encontrados (1.000, 10.000 e 13 vezes, respectivamente), enquanto a alfa-fetoproteína estava normal, indicando um tumor maligno no ducto biliar, não oriundo do fígado. Trinta e seis horas após a hospitalização, a paciente evoluiu a óbito. Os achados necroscópicos incluíram hepatite aguda com colapso hepatocelular, bem como metástase de carcinoma, provavelmente do ducto biliar.

**CONCLUSÃO:**

Apesar do carcinoma apresentado pela paciente, o uso de nimesulida pode ter contribuído para o dano hepático. Até que esta questão seja esclarecida, a prescrição de nimesulida deve ser cuidadosa para pacientes com doenças hepáticas.

## INTRODUCTION

Nimesulide (NIM) has analgesic, anti-inflammatory and anti-pyretic activity due to its potent inhibitory effects on the enzyme cyclooxygenase 2 (COX-2), while causing relatively low occurrence of gastrointestinal injury.^1^ Despite being widely prescribed and considered quite safe, nimesulide has been linked to cases of severe liver injury, especially when prescribed to susceptible patients.^2^^,^^3^^,^^4^^,^^5^^,^^6^^,^^7^ The hepatic events that have been reported during treatment with nimesulide include asymptomatic and reversible elevation of liver enzymes,^8^^,^^9^ acute hepatitis associated with hepatocellular necrosis, cholestasis and some isolated cases of fatal acute liver failure.^10^^,^^11^

Nimesulide-induced fatal hepatotoxicity is uncommon (one case in one million patients), even though nimesulide is one of the non-steroidal anti-inflammatory drugs (NSAIDs) that more frequently induce acute hepatitis.^4^^,^^12^ It is relatively insensitive to accumulated dose and is patient-specific, and it is considered to have idiosyncratic toxicity.^7^ In a analyses on 32 cases of liver failure of unknown cause after transplantation, six were correlated with therapeutic nimesulide ingestion within six months.^3^ In a retrospective study on drug-induced liver injury, Licata et al.^13^ found that nimesulide corresponded to 70% of all NSAID-related cases. Moreover, it has been reported that NIM-induced hepatotoxicity seems to be more common in middle-age females.^3^^,^^14^^,^^15^^,^^16^ Interestingly, in a case report on six cases of NIM-induced hepatotoxicity, Van Steenbergen et al.^10^ reported hepatocellular necrosis in all four female patients, whereas they demonstrated bland cholestasis in the remaining two male patients.

Experimental evidence shows that, besides hepatotoxicity, nimesulide promotes hyperplasia of the bile ducts and formation of reactive metabolites that covalently modify proteins, and it induces oxidative stress and mitochondrial injury.^17^^,^^18^ In NIM-induced cell damage, reduced glutathione consumption has been observed, with consequent oxidative stress and damage to the macromolecular infrastructure, thereby leading to mitochondrial uncoupling, decreased phosphorylation index (ADP/O_2_ ratio), and mitochondrial membrane permeability transition, followed by cell death.^11^^,^^17^^,^^18^^,^^19^^,^^20^

The individual risk factors that can predispose a patient to increased risk of developing nimesulide-associated hepatic adverse reactions, such as specific gene abnormalities, alterations to specific gene expression or epigenetic factors, are currently unknown.^7^ We report on the case of an elderly female with hepatic adenocarcinoma metastasis from the biliary duct, with early onset of hematological disorders after ingestion of nimesulide at therapeutic doses.

## CASE REPORT

A 81-year-old female with no hepatitis, no medical history of adverse drug reactions and adequate renal and hepatic function developed hematemesis and epistaxis two days before hospitalization in a university hospital in southern Brazil. She had taken NIM, once a day, for six days. Three weeks earlier, she had taken diclofenac for seven days, which was suspended four days before the beginning of nimesulide therapy.

On medical admission, large amounts of diffuse hematomas, bilateral epistaxis and melena were observed. The patient presented hypotension (80 x 40 mmHg), heart rate of 70 beats per minute, respiratory frequency of 26 breaths per minute, spontaneous ventilation and sudoresis. The laboratory tests on admission showed metabolic acidosis compensated with respiratory alkalosis, and increased lactate. Prothrombin time (PT), partial thromboplastin time (PTT), fibrinogen level and platelet count were low and therefore fresh-frozen plasma and platelet transfusion was conducted. The hematological profile comprised diminished red blood cell, hemoglobin and hematocrit levels, with leukocytosis. The renal profile on admission was characterized by increased urea and creatinine levels. Serum aspartate transaminase (AST), an indicator of deep hepatic lesions, was ten times above the normal level, while serum alanine transaminase (ALT) levels were normal. Total and direct bilirubin levels were increased but indirect bilirubin was normal. C-reactive protein, an inflammatory biomarker, was 30 times above the normal level. To rule out the suspicion of upper gastrointestinal bleeding, upper gastrointestinal endoscopy was conducted. During this examination, the patient suffered respiratory arrest and evolved to a decreased consciousness level, needing orotracheal intubation.

Eighteen hours after admission, the renal and hepatic damage advanced, as demonstrated by higher levels of urea, creatinine, AST, ALT and bilirubins (including the indirect form). At this time, gamma-glutamyl transferase (GGT) and alkaline phosphatase were determined and their levels were highly elevated. The hematological profile continued to worsen and, 36 hours after hospitalization, a red blood cell, fresh-frozen plasma and cryoprecipitate transfusion was conducted. On the same day, the hospital’s poison information center was notified in order to investigate the possibility of intoxication.

All the laboratory test results are presented in Table 1. Testing for hepatitis C and B produced negative results. Carcinoembryonic antigen (CEA) and CA-19-9 levels were increased, respectively, by 1,000 and 10,000-fold. CA-125 was slightly increased (13-fold) and alpha-fetoprotein (AFP) levels were normal (Table 2). Soon after the blood component transfusion, the patient’s general state continued to worsen and she suffered a 12-minute cardiorespiratory arrest which evolved to asystole and death.

**Table 1.**
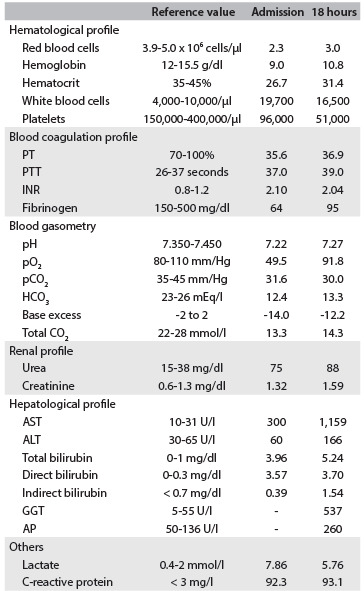
Laboratory tests on admission (0 hour) and 18 hours after hospitalization

Hematological, blood coagulation, renal and hepatological profiles and blood gasometry, lactate and C-reactive protein in an 81-year-old woman with acute hepatitis induced by nimesulide therapy. PT = prothrombin time; PTT = partial thromboplastin time activity; INR = international normalization ratio; AST = aspartate transaminase; ALT = alanine transaminase; GGT = gamma-glutamyl transferase, AP = alkaline phosphatase.

**Table 2.**
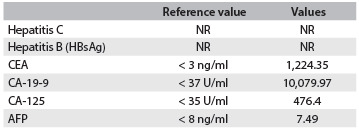
Hepatitis and cancer biomarker tests

Hepatitis C and B tests and CEA, CA-19-9, CA-125 and AFP cancer biomarkers in an 81-year-old woman with acute hepatitis induced by nimesulide therapy. Increased CEA together with increased CA-19-9 and CA-125 indicates a malignant tumor originating from the bile duct. Normal AFP levels rule out malignant tumors originating from the liver. CEA = carcinoembryonic antigen. AFP = alpha-fetoprotein. NR = non-reactive.

**Figure 1.**
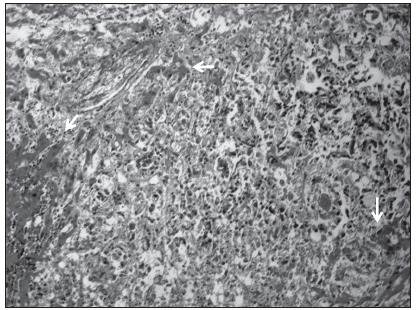
Histopathological features of the liver, focusing on acute liver failure: acute hepatitis with hepatocellular collapse in the patient’s liver. The arrows show extensive areas of coagulative necrosis and inflammatory infiltrate (hematoxylin and eosin stain; original magnification X 20).

**Figure 2.**
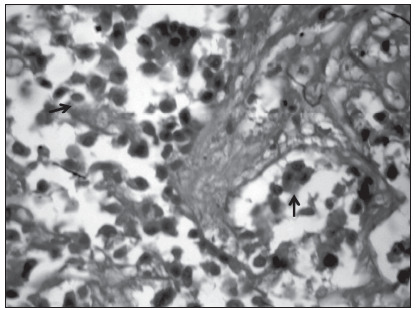
Histopathological features of the liver, focusing on adenocarcinoma metastasis: adenocarcinoma metastasis, probably originating from the bile duct, in the patient’s liver. The arrows show the neoplastic cells (hematoxylin and eosin stain; original magnification X 40).

The necropsy findings included liver acute hepatitis with inflammatory infiltrate, coagulative necrosis and hepatocellular collapse (Figure 1). Moreover, there was metastasis of moderately differentiated carcinoma, probably from the bile duct (Figure 2).

## DISCUSSION

To the best of our knowledge, this is the first case report on NIM-induced hepatotoxicity in a liver cancer patient, and in a Brazilian subject. Cancer is a major public health problem and is responsible for 27% of deaths worldwide, surpassed only by cardiovascular diseases. Its prevalence is increasing in developing countries as a result of population aging.^21^ Females over 50 years of age seem to be highly susceptible to NIM-induced liver damage, which generally presents rapid worsening, despite cessation of drug therapy.^3^^,^^13^^,^^14^^,^^16^

We conducted a systematic search in PubMed (Medline), Scopus, Cochrane Library, Lilacs and SciELO, to find papers that reported human hepatic injury due to NSAIDs (Table 3). Nimesulide is one of the NSAIDs that more frequently induce hepatic injury, and the majority of cases were correlated with therapeutic drug use. Furthermore, nimesulide-induced hepatotoxicity seems to be more common in middle-age females. The hepatic events reported during treatment with nimesulide include from asymptomatic and reversible elevation of liver enzymes to fatal acute liver failure. Although the patient had taken diclofenac three weeks before developing the clinical symptoms, we believe that nimesulide played a major role in her liver injury: first, because the patient was within the at-risk group; and second, because nimesulide was the current NSAID therapy. Several case reports have described occurrences of acute hepatitis with inflammatory infiltrate and hepatocellular collapse in cases of NIM-induced toxicity.^4^^,^^10^^,^^14^^,^^16^ In this case report, this condition was confirmed biochemically (increased AST, ALT, GGT and bilirubin levels) and histopathologically. Although the patient did not present jaundice, total bilirubin was higher than 3 mg/dl, with predominance of the direct form. When associated with increased alkaline phosphatase, the increase in direct bilirubin indicates post-hepatic hyperbilirubinemia, which can occur due to bile duct obstruction or biliary tract tumors.^22^^,^^23^ In NIM-induced liver failure, cholestatic liver injury is also reported, and this could explain the predominance of direct bilirubin and the increased alkaline phosphatase levels.^4^^,^^10^ The extensive liver damage observed explains the coagulation disorders, which are a common feature in nimesulide toxicity,^6^^,^^16^ as well as the metabolic acidosis due to increased lactate.^23^ It is known that solid tumors and metastasis can induce disseminated intravascular coagulation (DIC),^24^^,^^25^ but no laboratory tests that could indicate this diagnosis were performed.

**Table 3.**
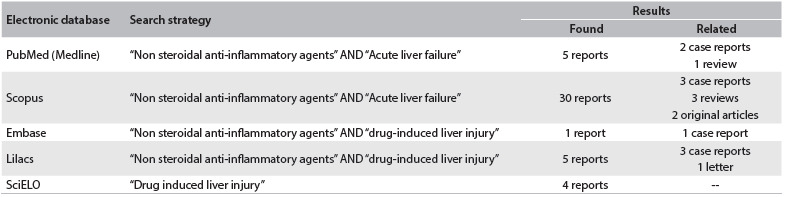
Literature review using MeSH (Medical Subject Headings) terms relating to human hepatic injury and use of non-steroidal anti-inflammatory agents, performed on September 18, 2013

Even though kidney failure was not observed at the necropsy, the patient presented some degree of renal damage, as suggested by the increased serum urea and creatinine levels. Schattner et al.^6^ described kidney failure in a 70-year-old woman who therapeutically ingested nimesulide for five days, two weeks before symptoms. Apostolou et al.^26^ reported on the case of a 68-year-old female who took one tablet of 100 mg of nimesulide and developed acute renal failure with diffuse ischemic lesions and lymphocyte infiltration into the kidney, similar to what has been described in cases of nephritis due to NSAIDs.

Cancer biomarkers are widely used for diagnosis, prognosis and patient follow-up.^27^ Approximately 99% of malignant gallbladder tumors originate from the epithelium, and 95% are adenocarcinomas.^28^ Increased CEA together with increased CA-19-9 is widely used as a gallbladder bile tumor marker.^29^^,^^30^ Simultaneous CEA, CA-19-9 and CA-125 elevation indicates primary malignancy from the bile ducts.^30^^,^^31^ Normal alpha-fetoprotein levels rule out cancer originating from the liver.^30^^,^^31^ Furthermore, following acute liver injury with extensive necrosis, an increase in AFP is interpreted as a sign of dedifferentiated hepatic regeneration.^32^^,^^33^^,^^34^ At the necropsy on the liver, the presence of both acute hepatitis and adenocarcinoma was confirmed, and the characteristics were those of a bile duct cancer metastasis. It is known that cancer can affect the function of the tissue in which it is hosted. Although the patient was in the group at risk of NIM-induced hepatotoxicity, the liver cancer metastasis probably also contributed towards the fatal acute liver failure.

## CONCLUSIONS

Based on the literature and the present case, until this issue has been clarified, caution is required in prescribing nimesulide for patients within the at-risk group, including liver disease patients.
